# Supplementary material for: Comparative Physiological Responses of Lemna aequinoctialis and Spirodela polyrhiza to Mercury Stress: Implications for Biomonitoring and Phytoremediation
Source: Plants (Basel). 2025 Sep 13;14(18):2859. doi: 10.3390/plants14182859 (PMC12473370; doi:10.3390/plants14182859)
Supplement: Supplementary file 1 [file plants-14-02859-s001.zip › plants-3759098-Supplementary Materials.pdf]

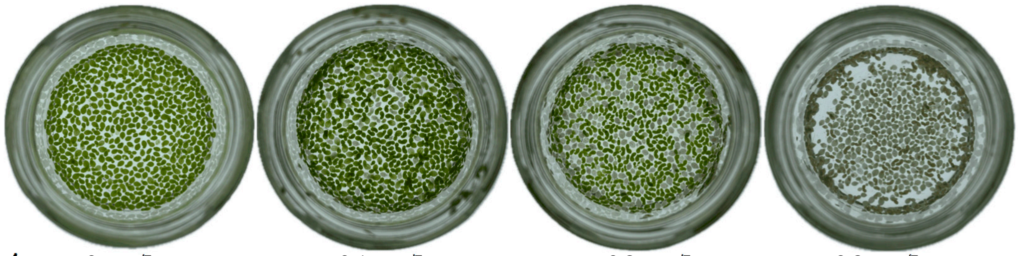

A 0 mg/L 0.1 mg/L 0.2 mg/L 0.3 mg/L

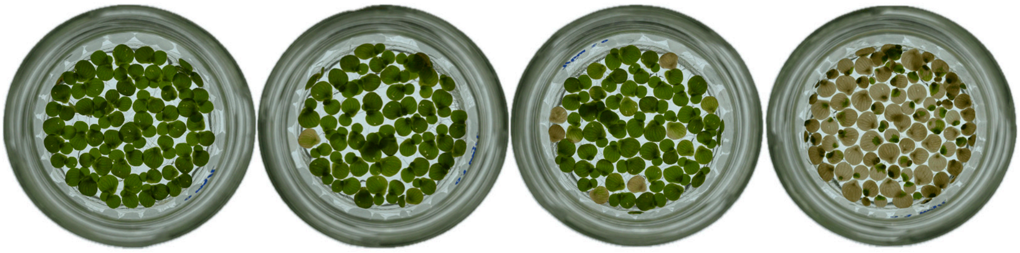

B 0 mg/L 0.1 mg/L 0.2 mg/L 0.3 mg/L

Figure S1. Images of frond in *L. aequinoctialis* **(A)** and *S. polyrhiza* **(B)** under different concentrations of mercury after 7 days.
